# Re-irradiation for recurrent glioblastoma: a pattern of care analysis

**DOI:** 10.1186/s12883-024-03954-z

**Published:** 2024-11-26

**Authors:** Susanne Rogers, Markus Gross, Ekin Ermis, Gizem Cosgun, Brigitta G. Baumert, Thomas Mader, Christina Schroeder, Nicoletta Lomax, Sara Alonso, Adela Ademaj, Tessa Lazeroms, Seok-Yun Lee, Michael Mayinger, Christoph Mamot, Lucia Schwyzer, Gerrit A. Schubert, Oliver Riesterer

**Affiliations:** 1 Radiation Oncology Center Mittelland, Kantonsspital Aarau, Aarau, Switzerland; 2 Department of Radiation Oncology, University Hospital Basel, Basel, Switzerland; 3 Department of Radiation Oncology, Inselspital, University Hospital Bern, Bern, Switzerland; 4 Department of Radiation Oncology, Kantonsspital Graubünden, Chur, Switzerland; 5 Department of Radiation Oncology, Kantonsspital Winterthur, Winterthur, Switzerland; 6 Department of Neurology, Kantonsspital Aarau, Aarau, Switzerland; 7 Department of Medical Oncology, Kantonsspital Aarau, Aarau, Switzerland; 8 Department of Radiation Oncology, University Hospital Zurich, Zurich, Switzerland; 9 Department of Neurosurgery, Kantonsspital Aarau, Aarau, Switzerland; 10 Department of Neurosurgery, RWTH Aachen University, Aachen, Germany

**Keywords:** Glioblastoma, Re-irradiation, Stereotactic, Moderate, Hypofractionated, Radiotherapy, Recurrence

## Abstract

**Background:**

90% of glioblastomas (GBM) relapse within two years of diagnosis. In contrast to the initial setting, there is no standard management for recurrent disease and options include hypofractionated stereotactic re-irradiation (re-mHSRT). The aims of this study were to investigate re-mHSRT practice in Swiss neuro-oncology centres.

**Methods:**

A survey of 18 questions regarding re-irradiation for GBM was created and distributed electronically (SurveyMonkey, USA) to 11 radiation oncologists in Switzerland specialising in brain tumours. We evaluated the clinical outcomes of a multicentre series of patients treated with an established re-mHSRT schedule to benchmark these against the literature and investigated the radiological patterns of relapse after re-mHSRT.

**Results:**

8 of 11 (73%) radiation oncologists responded to the survey and re-irradiation practice was heterogeneous. The 10 × 3.5 Gy schedule (RTOG 1205, BRIOChe trials) was used by 5/8 respondents and 47/50 patients with recurrent GBM treated with re-mHSRT with this schedule in daily practice were included in the analysis. The median time to re-mHSRT following completion of adjuvant RT was 23.3 (7-224) months. The median PTV at re-mHSRT was 22.0 cm^3^ (0.9–190). Combined CTV + PTV margins ranged from 0 to 10 mm and median prescription isodose was 80% (67–100). 14/47 (30%) patients received temozolomide and four (8.5%) continued bevacizumab concomitantly. On multivariable analysis, concomitant systemic therapy predicted for progression-free survival (PFS), HR 2.87 (95% CI 1-03-7.96), *p* = 0.042. Median PFS following re-mHSRT was 6.6 (0.2–92.5) months and 26/47 patients (55%) received subsequent systemic therapy. The median overall survival (OS) following recurrence was 11.8 months (1.5–92.5), similar to the 10.8 months in the literature with the same schedule. The six-month OS rate was 37/47 (79%), which compares well with the 73% reported in a meta-analysis of 50 publications employing various schedules. In a subgroup analysis of 36/47 (79%) patients with MR follow-up after re-mHSRT, 8/36 (22%) had no radiological evidence of tumour progression at a median follow-up of 9.4 months. 21/28 (75%) radiological relapses were in-field, two were marginal and five were out of field.

**Conclusions:**

Re-mHSRT with 10 × 3.5 Gy can achieve local control in selected patients with recurrent GBM.

**Supplementary Information:**

The online version contains supplementary material available at 10.1186/s12883-024-03954-z.

## Background

Despite advances in neuroradiology, neurosurgery, radiotherapy (RT) and the addition of temozolomide (TMZ) and tumour-treating electrical fields (Optune©, Novocure, Switzerland) to the therapeutic armamentarium, 90% of glioblastomas (GBM) relapse within two years of diagnosis. In contrast to the initial setting [1], there is no standard management for recurrent disease and options include re-irradiation using a moderately fractionated stereotactic technique (re-HSRT). Unlike initial postoperative RT, where margins of up to 2 cm may be added to cover potential microscopic spread [2], re-HSRT typically uses 0–3 mm margins due to concerns regarding the increased risk of brain necrosis. As re-irradiation becomes more frequent in clinical practice, outcome and toxicity data are helping to establish best practice, and an ESTRO/EORTC endorsed consensus statement as to definitions, reporting and clinical decision making for re-irradiation has recently been published [3]. Re-irradiation is at the forefront of radiation research as questions regarding optimal technique, fractionation schedules and normal tissue tolerance remain.

Current practice is heterogeneous as single fraction radiosurgery through to twenty fraction schedules are reported for the re-irradiation of GBM. A 10 × 3.5 Gy daily schedule [4] has been recently reported in the RTOG 1205 phase II trial of bevacizumab compared with bevacizumab plus re-irradiation with 10 × 3.5 Gy [5]. It forms the experimental arm of the open UK BRIOChe trial [6] and thus seems to have international acceptance. The aims of this study were to evaluate current practice in Swiss neuro-oncology centres and to identify the clinical outcomes of a real world series of patients treated with the 10 × 3.5 Gy schedule (subsequently referred to as moderately hypofractionated stereotactic radiotherapy or mHSRT) and to benchmark these against the re-mHSRT literature. Furthermore, we investigated the radiological patterns of relapse after re-mHSRT which have not been well described.

## Materials and methods

A survey consisting of 18 questions regarding re-irradiation for GBM was created and distributed electronically (SurveyMonkey, USA) to 11 radiation oncologists in Switzerland specialising in radiotherapy for brain tumours. Colleagues who reported using the 10 × 3.5 Gy fractionation and did not withhold their name were contacted and asked to contribute partially anonymised patient data. Ethics committee approval for the multicentre analysis was obtained (EKNZ 2023 − 00414).

Patients included in the analysis were treated with a stereotactic radiotherapy technique. Radiological outcomes were assessed on 3 monthly serial Gd_T1 MPR and FLAIR MRI images. Clinical endpoints were progression free survival (PFS), overall survival (OS) and toxicity. The survival outcomes were analysed using the Kaplan Meier estimator.

Multivariate analysis using Cox regression was performed to investigate the association PFS with the following factors: MGMT methylation status, Combs prognostic score, re-resection prior to re-mHSRT, concomitant systemic therapy. The analysis was performed in R (version 4.2.3) using the “survival” and “survminer” packages.

## Results

### Survey

8 of 11 (72.7%) radiation oncologists responded to the survey. The technical details of re-mHSRT as delivered in the eight centres are summarised in Additional File 1. The 10 × 3.5 Gy schedule was used by 5/8 respondents, although there were differences regarding clinical target volume margins, planning target volume (PTV) margins and prescription point or isodose.

50 patients with recurrent high-grade glioma were treated with re-mHSRT with 10 × 3.5 Gy between 07/2013 and 03/2023. Three were lost to follow-up, thus 47 patients were included in the analysis of clinical outcomes. Four patients had grade 3 glioma and were included in the analysis, as per the RTOG 1205 trial. Patient characteristics are summarised in Table 1.

**Fig. 1:**
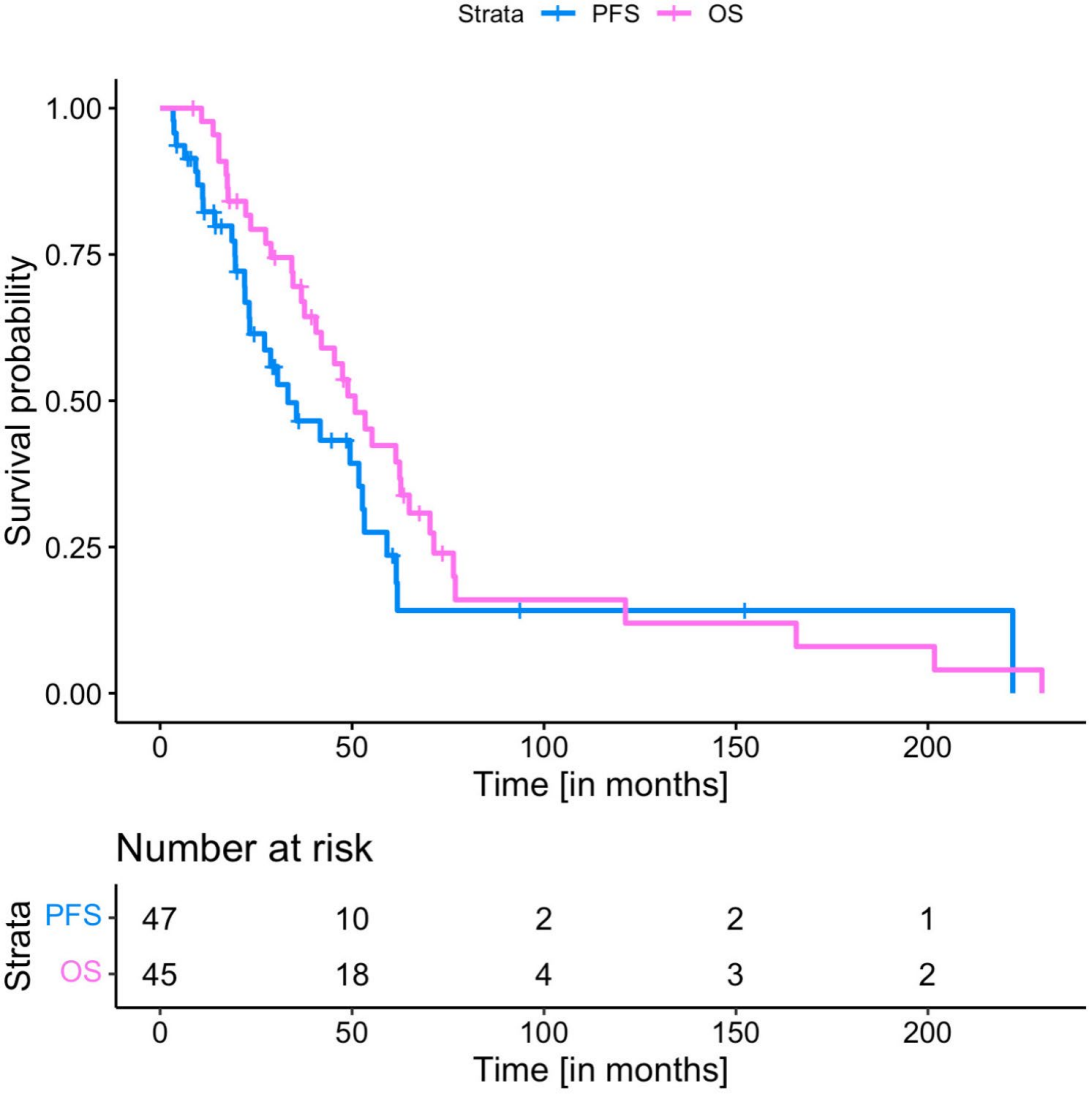
Kaplan-Meier curves of progression free survival (PFS) and overall survival (OS) (calculated from date of recurrence)

The median time to re-mHSRT following completion of adjuvant RT was 23.3 (7-224) months. The median PTV at re-mHSRT was 22.0 cm^3^ (0.9–190). The median combined CTV + PTV margins was 2 mm (range 0 to 10 mm) and the median prescription isodose was 80% (67–100). 14/47 (30%) patients received TMZ and four (8.5%) continued bevacizumab concomitantly. The median PFS following re-mHSRT was 6.6 (0.2–92.5) months (Figs. 1) and 26/47 (55%) received subsequent systemic therapy. The median overall survival (OS) following recurrence was 11.8 months (1.5–92.5). Median OS from the start of re-mHSRT was 8.8 (1-92.5) months and the six-month OS rate was 37/47 (79%) (Fig. 1). Toxicities were not systematically prospectively documented.

The multivariable analysis revealed that combining concomitant systematic therapy with re-mHSRT was associated with duration of PFS (hazard ratio (HR): 2.87, 95% CI: 1.03–7.96, *p* = 0.042). However, MGMT methylation status, Combs prognostic score and re-resection prior to re-mHSRT were not associated with PFS (Table 2). Furthermore, none of these factors were associated with OS (Table 2).

In a subgroup analysis of 36/47 (79%) patients with MR follow-up after re-mHSRT, 8/36 (22%) had no radiological evidence of tumour progression at a median follow-up of 9.4 months. 21/28 (75%) radiological relapses were in-field, two were marginal and five were out of field. All recurrences were diagnosed on Gd_T1 MRI with perfusion MRI and several patients also had a FET-PET to differentiate from pseudoprogression. Radionecrosis was diagnosed in four patients at a median interval of 9.9 months (range 6.47–10.83) after re-mHSRT. In a single institution series of fifteen patients, the initial diagnostic Gd_T1 MRI was available for ten patients and fusion with the Gd_T1 MRI at recurrence showed that eight (80%) first recurrences were in-field, having received a mean dose of 101.2% (97.1-103.3%) of the initial prescription, one was marginal (Dmean 90.1%, Dmin 56.9%, Dmax 104.3%) and one was distant (Dmean 33.9%, Dmin 24.7%, Dmax 59.7%) [7]. The out of field recurrence did not show a ‘leading edge’ on FLAIR MRI prior to development of the new contrast-enhancing lesion five months after initial RT, nor did the marginal recurrence develop within an initial FLAIR signal.

Patients were classified into three groups according to the ‘modified new Combs prognostic score’ (a = 0, b = 25, c = 18, d = 4) (Table 1) [8]. Thirty-nine patients had previously received maximum safe tumour resection followed by 30 × 2 Gy or 33 × 1.8 Gy with concomitant TMZ [1]. Two patients had received tumour treating fields (TTF) therapy [9]. Six patients over 70 years old received 15 × 2.67 Gy [10], all with TMZ according to the methylated MGMT status [11]. Following re-mHSRT, the cumulative normal tissue doses to brain (EQD2 α/β = 2) following 60 Gy and 40.05 Gy were 108.13 Gy and 94.83 Gy respectively. Nineteen patients underwent re-resection and 21/47 (45%) had had at least one second-line systemic therapy prior to re-mHSRT. Bevacizumab had been prescribed to 10/21, temozolomide to 8/21. Three patients had received lomustine, two in combination with bevacizumab. At progression after re-mHSRT, 28/47 (60%) patients received further systemic therapy.

**Table 1:** Multicentre analysis of 47 patients re-irradiated with 10 × 3.5 Gy

Patient Characteristics	*n* = 47
Male : Female	35 : 12
Age (years), median (range)	61.5 (23–81)
Karnofsky performance status, median (range)	90 (60–100)
WHO glioma classification 3:4	4: 43
Combs’ modified prognostic scores b: c:d	25: 18: 4
IDH WT : mutant : unknown	37 : 6: 4
MGMT methylation Y: N: unknown	24: 15: 8
Symptoms Y: N	30: 17
Initial 30–33 × 1.8–2 Gy : 15 × 2.67 Gy : other	39: 6: 2
Median PTV at re-mHSRT (cm^3^)	22.0 (0.9–190)
Re-resection prior to re-mHSRT Y : N	20 : 27
Second line systemic therapy prior to re-mHSRT Y : N	21 : 26 9 temozolomide 9 bevacizumab 1 bevacizumab/lomustine 1 lomustine 1 experimental agent
Concomitant systemic treatment with re-mHSRT Y: N	18 : 29 14 temozolomide 4 bevacizumab 29 none
Systemic treatment following re-mHSRT Y: N:unknown	28 : 18: 1 10 becavizumab 9 temozolomide 6 bevacizumab/lomustine 2 lomustine 1 surgery and re-mHSRT
Radionecrosis (*n* = 45/47 evaluable)	4 (8.8%)
Time to re-mHSRT after first RT (mths) median, range	23.2 (3.4–224)
Time to progression after first RT (months) median, range	23.3 (7.0-224)
Time to progression after re-mHSRT (*n* = 36) (months) median, range	6.6 (0.23–92.5)
Overall survival from start of re-mHSRT (months) median, range	8.8 (1.50–92.5)
Overall survival after first progression (months) median, range	11.8 (2.30–94)
Overall survival from first diagnosis (months)	40.6 (8.6 = 229.7

IDH = isocitrate dehydrogenase, MGMT = O-6-Methylguanine-DNA-Methyltransferase

ATRX = alpha-thalassemia/mental retardation syndrome X-linked, re-

mHSRT = moderately hypofractionated stereotactic re-irradiation

**Table 2:** Results of multivariable Cox regression analyses for progression free survival and overall survival

Progression free survival			Overall survival	
	HR (95%CI)	p	HR (95%CI)	p
Combs modified score a: b:c: d	1.39 (95%CI 0.32–1.90)	0.381	0.86 (95%CI 0.46–1.59)	0.631
MGMT status (yes/no)	0.79 (95%CI 0.32–1.96)	0.621	1.38 (95%CI 0.58–3.29)	0.468
Any systemic therapy concomitant with re-mHSRT (yes/no)	2.87 (95%CI: 1.03–7.96)	**0.042**	0.77 (95%CI 0.31–1.92)	0.582
Re-resection prior to re-mHSRT (yes/no)	1.36 (95%CI 0.55–3.34)	0.497	0.51 (95%CI 0.21–1.21)	0.122

**Table 3:** Summary of the key literature describing clinical outcomes with 10 × 3.5 Gy re-mHSRT to act as a benchmark for this series

Author	Grade 4 glioma/total patients (*n*=)	Age at recurrence (yrs) median, range	Median time to re-mHSRT (months)	Median OS from recurrence (months)	Median OS from diagnosis (months)	Rate of radionecrosis (%)	Adverse events (CTCAE grade)			Concomitant systemic treatment	Median planning target volume (cm^3^)	RE-FSRT dose** Gy/fractions	CTV margin (mm)	PTV margin (mm)
							3	4	5					
Fogh et al. 2010	105/147	53 (19–86)	8	11.0 (9, 14)	23 (18–26)	0.7	1	0	0	41% (various types of chemotherapy)	14 vs. 33 after surgery	35/10	0	0
Palmer* et al. 2015	208	54 (22–81)	9 (RT) vs. 11.6 (repeat surgery + RT)	10.5 (RT) vs. 11.1 (repeat surgery + RT)	22.5	NR	1	0	0	58% (mostly bevacizumab)	21 vs. 33 after surgery	35/10	0	0
Palmer* et al. 2018	87	57 (21–85)	10.8	11.9 (4.4, 18.3)	24.4 (9.7–94.2)	NR	7	0	0	Bevacizumab: before vs. after	21.4	35/10	0	0
Klobukoswski et al. 2018	62/76	56 (17–83)	NR	9.5 from re-mHSRT	29.1	4	3	0	0					
Tsien et al. 2023	170	NR	NR	10.1 (with BV) vs. 9.7 (without)	unknown	0	5%	0	0	Bevacizumab: concomitant or alone	53	35/10	max.5	3
This series	41/47	61 (23–81)	23.3	11.8 (2.30–94)	42 (8.6-1212.2)	8.8 (4/45)	NR	NR	NR	38% (mostly temozolomide)	22 (0.9–190)	35/10	CTV + PTV = 0–8	

NR = not reported, *may include repeat patients, ** same total dose but with variable prescription isodoses and normalization points, re-mHSRT = moderately hypofractionated stereotactic re-irradiation, BV = bevacizumab

## Discussion

Widely used clinical guidelines (NCCN, EANO) list multiple options without preferential weighting for the management of recurrent GBM from repeat surgery, re-irradiation and systemic therapy through to best supportive care. Given the guarded prognosis associated with relapsed GBM, each option needs to be carefully evaluated in terms of expected benefit and likely toxicities, for example with regard to tumour location and patient performance status. Prognostication is challenging and the use of validated scoring systems in the selection of patients for re-irradiation is recommended. The original validated Combs prognostic score consisted of histology, patient age and time to re-mHSRT, although multivariate analyses have yielded conflicting data regarding the prognostic significance of the interval between adjuvant and salvage radiotherapy. The Combs score was subsequently modified through the inclusion of Karnofsky performance status, PTV and whether a re-resection had been performed [8]. A good performance status and a smaller PTV are consistently reported to be positive prognostic factors and re-resection is associated with a better clinical outcome according to several systematic reviews [12, 13]. According to the modified Combs prognostic score, our patients grouped predominantly into categories b and c, which are associated with an expected median survival following re-mHSRT of 11.3 and 8.1 months, respectively. The observed survival in the 44/47 patients in these two categories in this series was 13.3 and 10.8 months respectively, suggesting this scoring system is useful for selecting patients with a better prognosis for re-irradiation. MGMT status is an accepted prognostic marker, however, was not included in the Combs scores as data was not available for all patients in the training cohort. A multivariable analysis of five clinical factors (MGMT methylation status, Combs score, re-resection, systemic therapy, and time to relapse) did not show any statistically significant effect on the duration of local control after re-mHSRT in our series, possibly because the patients were already highly selected.

Lomustine has become standard second line therapy in many European centres [14] whereas in the USA, bevacizumab is widely used in the relapsed setting [15, 16]. In a Cochrane meta-analysis, where lomustine was taken as the comparator, no data suggested any other therapy to be superior [17]. Outcomes from trials comparing re-mHSRT alone with concomitant systemic therapy are mixed. A retrospective study reported a doubling in 1 year PFS and OS when re-irradiation with stereotactic radiosurgery (SRS) was combined with bevacizumab as opposed to delivered alone [18] and a benefit was also seen from the combination with re-mHSRT [19]. The RTOG 1205 phase II trial examined the potential benefit of bevacizumab concomitantly with re-mHSRT at 10 × 3.5 Gy randomised against bevacizumab alone. The combination increased 6-month PFS from 29.1 to 54% however failed to increase median overall survival, which was 10.1 months as compared with 9.7 months in the control arm [5]. These OS data are similar to the 11 months reported by Fogh et al. following re-mHSRT alone with the same schedule [4]. The median OS from re-mHSRT in the Swiss patients was 8.8 months, close to the 9.5 months reported in a similar retrospective publication [20]. The PFS in the RTOG 1205 trial was 7.1 months with re-mHSRT and bevacizumab, as compared to 3.8 months with bevacizumab alone. The median PFS of 7.2 months and 6-month PFS of 63% in our Swiss re-mHSRT series with and without concomitant systemic therapies compares well.

The RTOG 1205 data echo the findings of the EORTC 26101 trial where the combination of bevacizumab and lomustine demonstrated a reduction in tumour volume and an increase in progression-free survival but failed to achieve a survival advantage compared to lomustine alone [21]. Moreover, the combination was associated with high rates of toxicity with 63.5% of patients experiencing grade 3 to 5 adverse events, which included pulmonary embolism, arterial hypertension and haematological toxicities [14]. The most relevant toxicity of lomustine is thrombocytopenia that often requires dose reduction and delays or even discontinuation of treatment [22], and bevacizumab is associated with thrombocytopenia, thromboembolic events and severe haemorrhage [23]. Despite the multiple active therapies available, more does not seem to be better. The 20% 6-month PFS observed with lomustine is taken to be the reference response rate for comparative trials [22]. 6-month PFS rates of 26% were observed with lomustine in MGMT methylated patients as compared with 0% in unmethylated patients in the BELOB trial of lomustine versus the combination of lomustine with bevacizumab [24]. Of the 39 patients in this series with known MGMT status at first or second surgery, 24 had a methylated gene promoter. Re-mHSRT may be relatively more beneficial to patients without MGMT methylation, where lomustine has low to no activity.

**Fig. 2:**
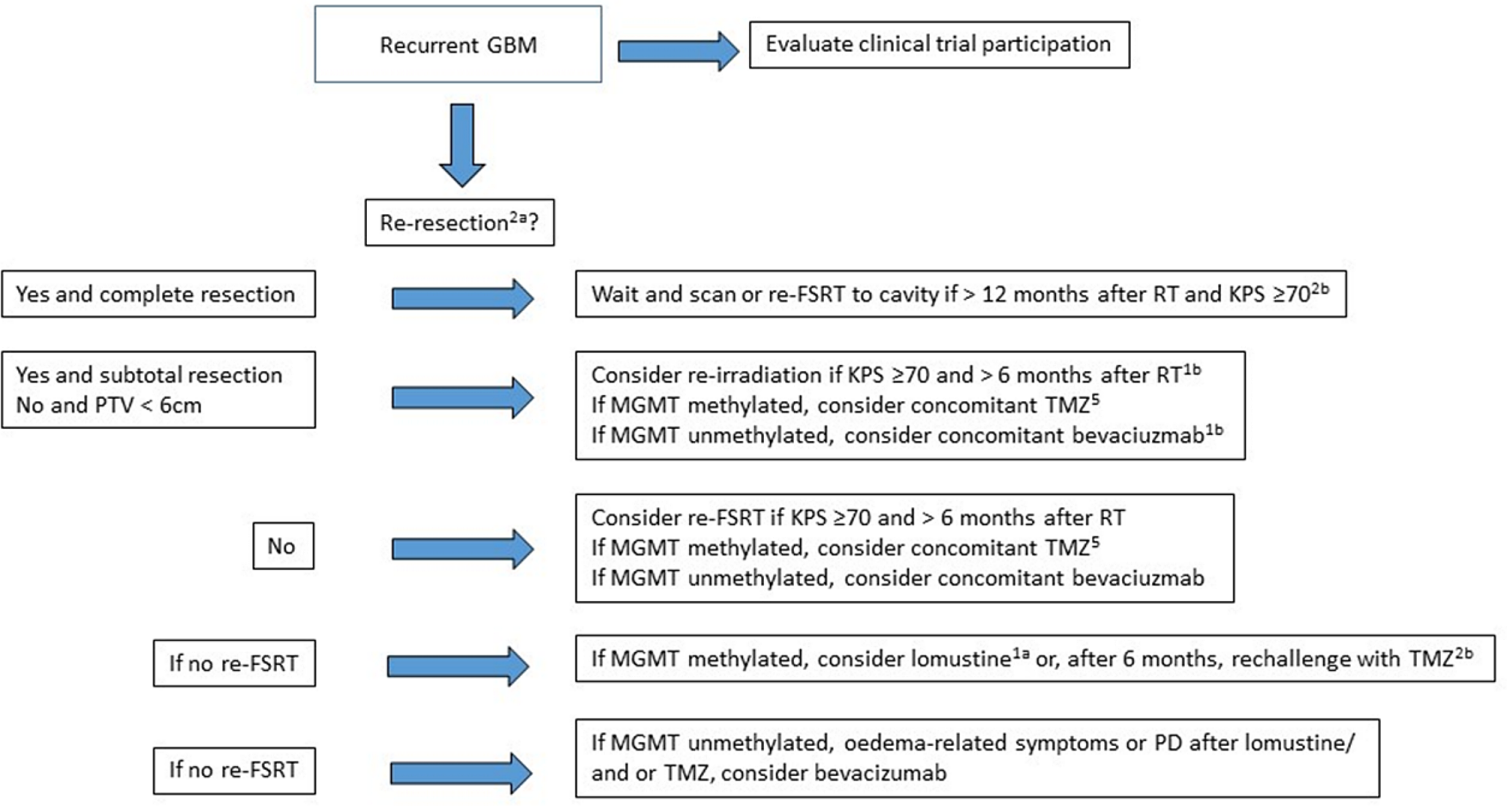
Flowchart representing considerations, annotated with level of evidence, underlying discussion and decision-making at a tumourboard for a patient with recurrent glioblastoma

Given the plethora of therapeutic options, with little evidence as to which might be preferable for a given patient at disease relapse, we have illustrated considerations which may underlie decision-making at the neuro-oncology tumourboard. MGMT status could be used to recommend either lomustine or a rechallenge with TMZ in MGMT methylated patients, or re-mHSRT in non-methylated small tumours at disease progression (Fig. 2). As the MGMT methylation status can change during the natural history of the disease, particularly in methylated patients [25], testing should be considered at re-resection if such a flowchart is to be followed.

Studies of the patterns of relapse can optimise and refine future therapy. There are two schools of thought regarding the need to include information from the T2-weighted image signal in the radiation volume. The RTOG contouring guidelines recommend two phase treatment planning, with inclusion of the oedema to 46 Gy and a boost without oedema to 60 Gy. In Europe, the convention is to treat the surgical cavity and contrast-enhancing residual tumour in one phase to 60 Gy, as it has been observed that 80% of cases relapse within 2 cm of the original tumour and thus within the 2 cm margins added to cover microscopic spread and technical uncertainties [26]. The main challenge with interpreting changes in the T2-weighted image is to distinguish between oedema and non-enhancing tumour and thus the FLAIR sequence is preferred over the T2-weighted image. Whereas oedema should not necessarily be included in the planning target volume [26], there is a local control and survival advantage in including non-contrast enhancing glioblastoma [27] and the CTV can be modified to include the FLAIR signal [26]. Duma et al. have proposed a radiosurgical boost of 10 Gy to the ‘leading edge’ on the FLAIR sequence, which is purported to represent the main path of microscopic spread [28]. Similarly, fractionated SRS to areas of FLAIR signal as well as regions with contrast enhancement has been suggested [29].

**Fig. 3:**
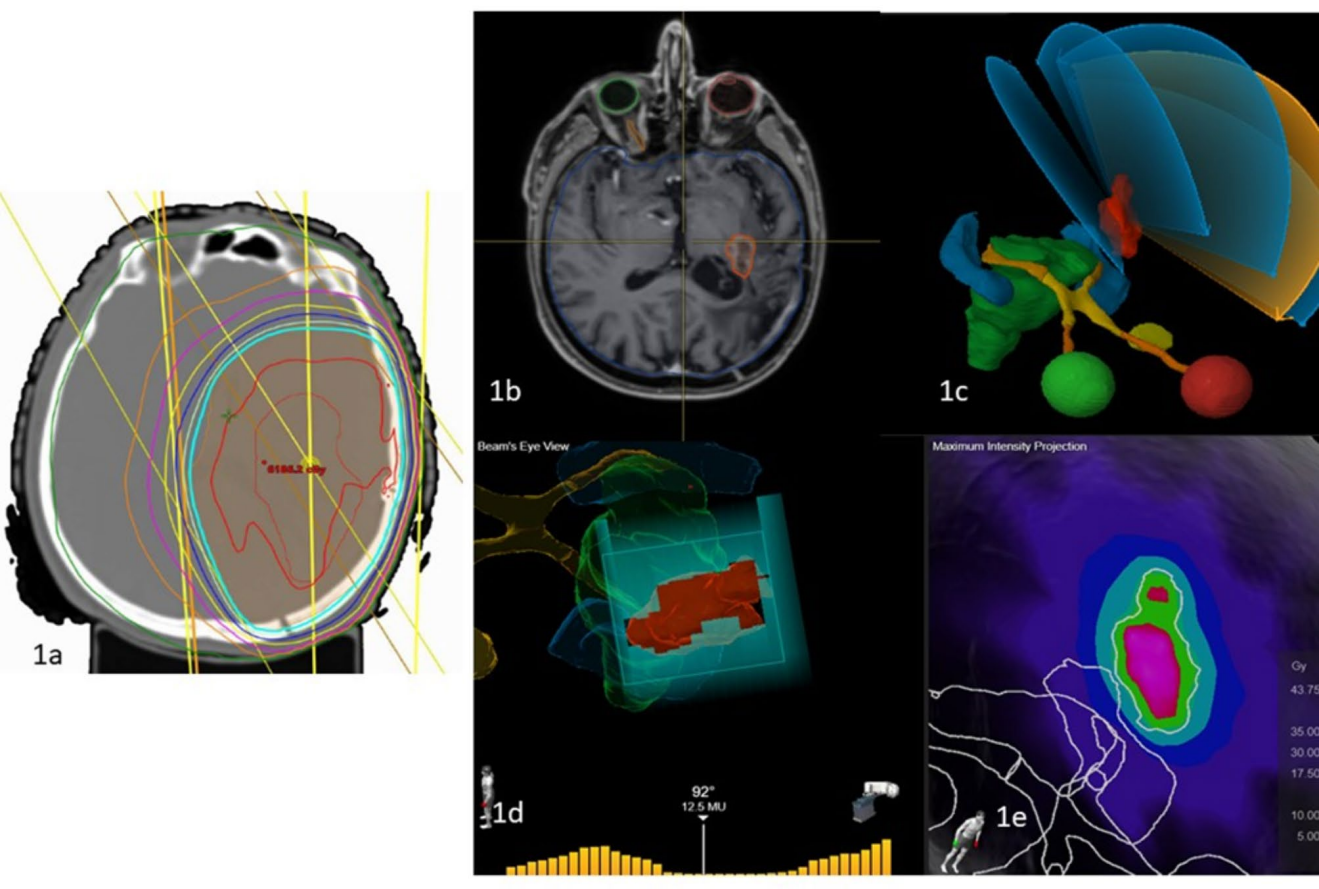
Representative patient re-irradiated with a stereotactic technique: 1a) 2018 Initial 60 Gy VMAT radiotherapy plan, 1b) 2022 in-field recurrent GBM with GTV contoured in orange, 1c) 5 non-coplanar arc VMAT plan, 1d) Beam Eye View (BEV) of one of the fields conforming to PTV (0 mm planning margin) with 2.5 mm MLCs, 1e) re-mHSRT dose distribution in 10 fractions (70% isodose dose surface: green 35 Gy, magenta 43.75 Gy)

We and Palmer et al. [19] used the method reported by Fogh et al. [4] without addition of margins for microscopic spread or technical uncertainties, whereas Fokas et al. [30] and Tsien et al. [5] added a 3–5 mm margin when using the same HSRT schedule. The administration of concomitant systemic therapy and different endpoint reporting confound any interpretation of the effect of the margin, however. Our practice, according to the ICRU 91 guidelines for SRS/HSRT [31], is that 99% of the PTV should receive 100% of the prescribed dose with a Dmax of 135-140% which is equivalent to prescribing to the 70 isodose dose surface (IDS) when normalised to the maximum dose (Fig. 3). The sharp dose fall-off is intuitive for maximal normal brain protection when treating demarcated brain metastases with SRS/HSRT, but less so for GBM which has an infiltrative growth pattern. Stereotactic re-irradiation is the recommended technique to spare previously irradiated normal brain and the median prescription isodose surface in the multicentre analysis was 80%, although some centres preferred a homogeneous dose prescription (100%). The margin concept varied accordingly, increasing from 0 mm to 2 mm to 8 mm as the prescription moved from a 70% IDS to an 80% IDS.

The pattern of first relapse after chemoradiation (CRT) in this series was, as expected, 80% within 2 cm of the surgical cavity [32]. On reviewing the marginal and distant relapses after CRT in a subgroup of 15 patients from one centre, these did not occur in regions of FLAIR signal. If re-mHSRT has a positive effect on clinical outcome, then the target volume definition, planning margins, dose delivered and prescription isodose should all be of significance. We therefore evaluated the location of the relapse after re-mHSRT given the open questions as to the adequacy of the 0 mm PTV margin, the effect of prior bevacizumab on target definition through the reduction of contrast-enhancement and the putative superiority of subtraction MRI over the Gd-T1 sequence. As 75% of the relapses occurred in-field, the 0 mm CTV/PTV margin with a 70% IDS, 2 mm margin with an 80% IDS and an 8 mm margin with a 100% dose prescription all seem to be valid approaches, and the margin width was non-significant on multivariable analysis. Reports of the pattern of failure after re-mHSRT are scarce, although Moore-Palhares et al. [33] recently made a similar observation with a 66% rate of in-field failure.

The local control rates of glioblastoma after surgery and radiotherapy remain unsatisfactory and optimising the initial target volume delineation is an active research area. Grosu et al. have published retrospective data regarding biology-guided target volume delineation using amino-acid PET-CT and the results of the prospective GLIAA (NOA-10, ARO 2103/1) trial comparing clinical outcomes with this methodology against the current standard CE-MRI are awaited. We and others have investigated diffusion tensor and fractional anisotropy maps for the identification of infiltrated white matter tracts for inclusion in the CTV [34, 35] however, at present, these approaches remain exploratory.

In the setting of recurrent GBM, contrast clearance analysis based on the subtraction of the background enhancement on a native sequence from a subsequent contrast-enhanced T1 sequence is reported to assist in the diagnosis of true progression versus pseudo-progression [36, 37]. It may also improve target definition in patients receiving bevacizumab, which reduces contrast enhancement and thus visualisation of the tumour, through normalisation of the vasculature [38]. As a subtraction sequence is widely available in centres with neuro MRI facilities, it offers practical advantages over radiomics-based techniques that require expert post-processing [39]. Our attempt to compare quantitatively the GTV delineated on CT-T1 MRI and retrospectively on the subtraction MRI proved unsuccessful however, because the subtraction was based on the 2D TE sequence, which does not provide the volumetric data set necessary for delineation of radiosurgical targets.

On multivariable analysis, concomitant systemic therapy was statistically significant for PFS, even though MGMT methylation status was not. Furthermore, Combs score, re-resection prior to re-mHSRT and time to first progression were all non-significant with regard to PFS and OS. The initial patient selection in our series may explain the lack of significance of these factors in our cohort. A recent retrospective analysis of 79 patients treated with re-mHSRT for high grade glioma [33] reported several factors associated with an increase in overall survival. For example, a PTV less than 112 cm3 was found to be favourable and, with a median of 22 cm3 (0.9–190), our patients had small target volumes in comparison. An interval from primary treatment to first progression in excess of 16.3. months was a positive prognosticator and again, our patients had a longer median time to first progression of 23.3 months (3.4–224). Numerous retrospective single centre series describing clinical outcomes following single fraction SRS and hypofractionated SRT (HSRT) have been recently reviewed by Minniti et al. [40]. To benchmark our practice, we compared our data with five series reporting outcomes following 10 × 3.5 Gy and observed very favourable results in terms of OS after re-mHSRT and toxicity (Table 3).

The weaknesses of our series are the small sample size due to the limited number of patients suitable for re-irradiation in the small Swiss population and the hetereogeneous fractionations in use. Furthermore, toxicity was not systematically recorded. The 4/47 patients with grade 3 glioma and additional 2 with IDH-mutant glioblastoma (WHO classification 2007/2016) would be expected to have a better prognosis and might have positively skewed the survival data, but are also the patients who might benefit the most from re-irradiation at relapse. The main strengths of the evaluation were that the patients included were treated with a standardised re-irradiation fractionation and that perfusion MRI [41] and FET-PET scans were frequently used to differentiate between true and pseudo-progression [42].

## Conclusion

Median PFS, OS and toxicity in our series compare favourably with those reported in the literature. The pattern of relapse after re-mHSRT for GBM was predominantly in-field, similar to that described after first irradiation. Systemic therapies, re-resection and re-irradiation of recurrent GBM are all reported to achieve overall survival of 6–12 months, but these do not appear to be cumulative in patients receiving a combination of two or three modalities. Re-mHSRT with 10 × 3.5 Gy appears to be a clinically meaningful option in selected patients with circumscribed recurrent GBM.

## Electronic supplementary material

Below is the link to the electronic supplementary material.

Supplementary Material 1

## Data Availability

Data will be shared if a reasonable Ethics-approved request is received.
