# Supplementary material for: Re-irradiation for recurrent glioblastoma: a pattern of care analysis
Source: BMC Neurol. 2024 Nov 26;24:462. doi: 10.1186/s12883-024-03954-z (PMC11590342; doi:10.1186/s12883-024-03954-z)
Supplement: Supplementary file 1 — Supplementary Material 1 [file 12883_2024_3954_MOESM1_ESM.docx]

| **Survey questions** | **Total centres, n= 8, (%)** |
| --- | --- |
| Do you offer re-irradiation at disease progression? | |
| Yes | 8/8 (100 %) |
| No | 0/8 (0 %) |
| Do you offer re-irradiation in, or analogous to, a clinical trial? | |
| Yes | 5/8 (62.5 %) |
| No | 3/8 (37.5 %) |
| Are patients for re-irradiation selected at a tumourboard? | |
| Always | 7/8 (87.5 %) |
| Usually | 1/8 (12.5 %) |
| How is recurrence generally diagnosed? | |
| T1 and perfusion MRI sequences | 6/8 (75 %) |
| FET-PET | 2/8 (25 %) |
| Do you follow any guidelines for re-irradiation? Please specify | |
| Yes | 6/8 (75 %) |
| No | 2/8 (25 %) |
| Do you regularly use a prognostic score? | |
| Yes | 0/8 (0 %) |
| No | 8/8 (100 %) |
| Do you consider MGMT methylation status to be an eligibility criterion for re-irradiation? | |
| Yes | 0/8 (0 %) |
| No | 8/8 (100 %) |
| Do you consider time interval from first radiotherapy to be an eligibility criterion for re-irradiation? | |
| Yes | 8/8 (100 %) |
| No | 0/8 (0 %) |
| Do you consider age to be an eligibility criterion for re-irradiation? | |
| Yes | 2/8 (25 %) |
| No | 6/8 (75 %) |
| Do you consider performance status to be an eligibility criterion for re-irradiation? | |
| Yes | 8/8 (0 %) |
| No | 0/8 (100 %) |
| Do you set a maximum tumour diameter/volume cut-off? | |
| Yes | 3/8 (37.5 %) |
| No | 5/8 (62.5 %) |
| Do you use a stereotactic immobilisation set-up? | |
| Yes | 8/8 (100 %) |
| No | 0/8 (0%) |
| Do you perform a 3-D contrast-enhanced planning MR scan? | |
| Yes | 8/8 (100 %) |
| No | 0/8 (0%) |
| Do you add a CTV margin? | |
| Yes, (in mm) | 3/8 (37.5 %), (5 - 10 mm) |
| No | 5/8 (62.5 %) |
| Do you add a PTV margin? |  |
| Yes, (in mm) | 7/8 (87.5 %), (2 - 5 mm) |
| No | 1/8 (12.5%) |
| Do you prescribe the dose to the isocentre (homogeneously)? | |
| Yes | 4/8 (50 %) |
| No, to which isodose? | 4/8 (50 %), (to the 70-80 % Isodose) |
| What machine do you use to deliver re-irradiation? | |
| Radiosurgery linac | 4/8 (50 %) |
| Conventional linac | 4/8 (50 %) |
| Do you usually offer re-irradiation to the cavity after gross total re-resection? | |
| Yes | 3/8 (37.5 %) |
| No | 5/8 (62.5 %) |
| Do you usually offer re-irradiation to the cavity after subtotal re-resection? | |
| Yes | 7/8 (87.5 %) |
| No | 1/8 (12.5 %) |
| Do you combine re-irradiation with temozolomide? | |
|  | 7/8 (87.5%) |
|  | 1/8 (12.5 %) |
| Do you combine re-irradiation with bevacizumab? | |
|  | 5/8 (62.5 %) |
|  | 3/8 (37.5 %) |

*****MGMT = O6-methylguanine-DNA methyl-transferase

**Supplementary Table 1** Summary of responses to the survey regarding current practice for re-irradiation in Switzerland
